# Breaking barriers: advancing cellular therapies in autoimmune disease management

**DOI:** 10.3389/fimmu.2024.1503099

**Published:** 2024-11-29

**Authors:** Yanhong Fu, Chunjing Feng, Shan Qin, Zhiyao Xing, Chong Liu, Zichuan Liu, Hongjian Yu

**Affiliations:** ^1^ School of Pharmaceutical Science and Technology, Faculty of Medicine, Tianjin University, Tianjin, China; ^2^ Tianjin University and Health-Biotech United Group Joint Laboratory of Innovative Drug Development and Translational Medicine, School of Pharmaceutical Science and Technology, Faculty of Medicine, Tianjin University, Tianjin, China; ^3^ Jiangxi Engineering Research Center for Stem Cell, Jiangxi Health-Biotech Stem Cell Technology Co., Ltd., Shangrao, Jiangxi, China; ^4^ Jinnan Hospital, Faculty of Medicine, Tianjin Jinnan Hospital, Tianjin University, Tianjin, China; ^5^ Frontiers Science Center for Synthetic Biology (Ministry of Education), Tianjin University, Tianjin, China

**Keywords:** autoimmune diseases, cellular therapy, B cells, CD19, Tregs

## Abstract

Autoimmune diseases occur due to a dysregulation within the immune system, leading to an aberrant assault on the organism’s own tissues. The pathogenesis of these conditions is multifactorial, encompassing intricate interplays among genetic predispositions, environmental determinants, and hormonal fluctuations. The spectrum of autoimmune diseases is broad, impacting a multitude of organ systems, with notable examples such as rheumatoid arthritis (RA), systemic lupus erythematosus (SLE), multiple sclerosis (MS), psoriasis, and vitiligo. Despite substantial progress in therapeutic interventions over recent years, a definitive cure for autoimmune diseases has yet to be realized, with existing modalities largely providing palliative care. Cellular therapy is considered the fourth pillar in the management of oncological disorders subsequent to surgical resection, radiotherapy, and chemotherapy. Cellular therapies have shown potential in augmenting immune competence and eliminating of targeted neoplastic cells in a spectrum of cancers. As targeting specific molecules on the surface of autoreactive B and T cells, such as CD19, BCMA, CD20, and CTLA-4, cellular therapies are emerging as promising approaches for the treatment of autoimmune diseases. This review delineates the advancements in the application of cellular therapies applied recently for autoimmune diseases and proposes considerations for the advancement of novel therapeutic strategies.

## 1 Introduction

The immune system, comprising immune organs, cells, and molecules, holds a pivotal position in the recognition and elimination of antigenic foreign entities, as well as in the coordination with other organic systems to maintain homeostasis and physiological equilibrium within the organism. However, once the immune system’s inability to discern between foreign antigens and self-host cells results in the development of classical or pathological autoimmunity, culminating in tissue damage and inducing autoimmune diseases (1). Autoimmune diseases, ranking as the third most common category of illnesses after cancer and cardiovascular diseases, affect an estimated 5-8% of the global population, with a combined prevalence of 10.2%—impacting 13.1% of women and 7.4% of men (2). Characterized by the disruption of immune tolerance, the etiology of autoimmune diseases is intricate, encompassing genetic, environmental, and hormonal factors (3). To date, over 100 distinct autoimmune diseases have been identified, with prevalent examples including rheumatoid arthritis (RA), systemic lupus erythematosus (SLE), and multiple sclerosis (MS) (4, 5). These conditions not only inflict substantial distress upon affected individuals but also pose a major global socio-economic challenge (6).

Reversing disrupted immune tolerance presents a significant challenge in the management of autoimmune diseases, with existing therapeutic strategies primarily aimed at disease control rather than cure (7). The advent of traditional synthetic disease-modifying antirheumatic drugs (DMARDs) and biologic DMARDs has led to substantial improvements in the prognosis of patients afflicted with autoimmune rheumatic diseases. Nevertheless, the majority of patients necessitate continuous drug therapy to ameliorate symptomatology. Nonspecific therapeutic approaches, predicated on the use of hormones and immunosuppressants, systematically suppress the immune system, thereby preventing autoreactive immune cells from assaulting host-body. This method, while effective, carries the risk of inducing severe adverse effects, including an increased susceptibility to infections (8). Biological agents, particularly monoclonal antibodies (mAbs) targeting TNFα and IL-6R, have demonstrated superior efficacy and reduced toxicity in comparison to conventional therapeutics. Despite these advancements, the necessity for repeated antibody infusions and the challenge of achieving a durable and effective restoration of immune homeostasis persist. Furthermore, the immunogenicity associated with the prolonged administration of antibodies poses an unresolved concern (9). Collectively, these therapeutic modalities often necessitate ongoing or intermittent drug administration, which is associated with the potential for cumulative long-term side effects. Most critically, they provide palliative relief and may slow disease progression, yet fail to reverse organ damage or physical disability (10).

With the continuous advancements in immunobiology and synthetic biology, along with the rapid progress of clinical-scale genetic engineering and gene editing technologies, various emerging cellular therapies have significantly advanced tumor therapy and other diseases (11). Cellular therapy has gradually become an important branch in the field of tumor treatment. It is mainly divided into two categories: native immune cell therapy and engineered immune cell therapy. Of native immune cell therapy, the patient’s own immune cells are primarily utilized, which are activated or expanded ex vivo and then reinfused into the patient to enhance the body’s immune response to tumor cells. Engineered immune cell therapy, on the other hand, involves genetically modifying immune cells to enable more precise recognition and destruction of tumor cells.

Targeted cellular therapies against autoreactive immune cells are considered a potential treatment for a series of autoimmune diseases. Chimeric Antigen Receptors (CARs), pioneered in the late 1980s, are synthetic transmembrane proteins with high specificity for target antigens (12). These receptors can redirect lymphocytes to recognize and exert their effects under specific conditions, offering non-major histocompatibility complex (MHC)-restricted recognition of cell surface components. Currently, various CAR T-cell therapies targeting different tumor antigens are either in clinical trials or have been approved for marketing. For instance, GD2 CAR T-cell therapy is specifically designed for neuroblastoma and other solid tumors that express the GD2 antigen (13). Mesothelin CAR T-cell therapy targets malignant pleural mesothelioma and other tumors expressing mesothelin (14). BCMA CAR T-cell therapy is primarily applied to patients with BCMA-positive multiple myeloma (15), and HER2 CAR T-cell therapy is directed against breast cancer and other tumors with HER2 overexpression (16, 17). Among these therapies, CD19 CAR T-cell therapy has garnered significant attention due to its remarkable efficacy in treating CD19+ B-cell hematological malignancies and has become one of the most successful cases in clinical application (18). Owing to its specificity and the induction of durable remission in autoimmunity, CAR cell therapy has been applied to the treatment of autoimmune diseases (19), with CAR-T cells, chimeric autoantibody receptor T cells (CAAR-T cells), and CAR-NK cell being used to deplete pathological immune cells, such as B cells, autoreactive B or T cells, and helper antigen-presenting cells (APCs), yielding favorable outcomes.

Furthermore, other cells are also under investigation for the treatment of autoimmune diseases, including regulatory T cells (Tregs), mesenchymal stem cells (MSCs) and hematopoietic stem cells (HSCs). This review discusses the advancements and challenges in cellular therapy for autoimmune diseases, providing insights for the development of novel therapeutic approaches targeting autoimmunity.

## 2 Autoimmune diseases

**Figure 1:**
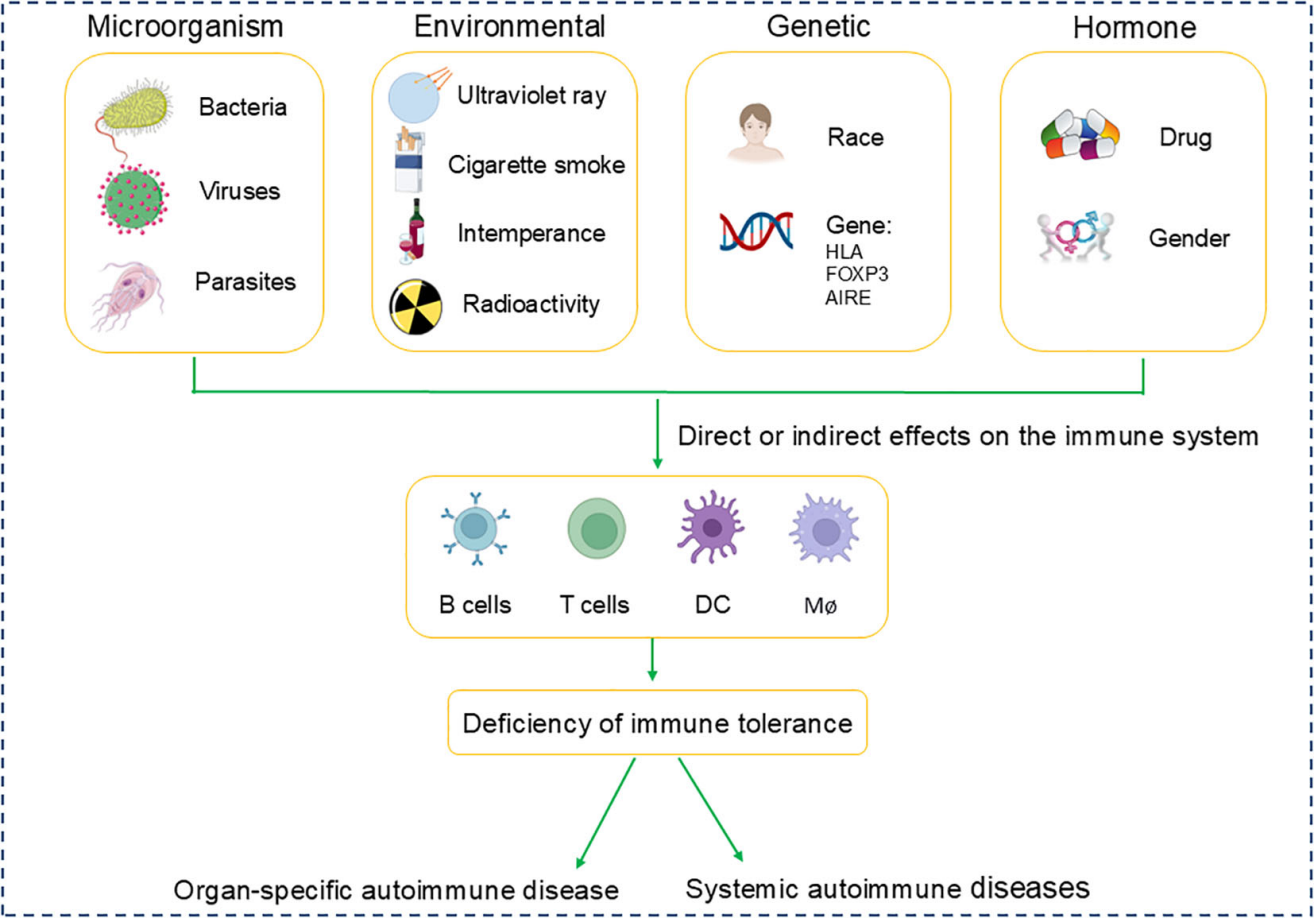
Pathogenic factors in autoimmune diseases.

The epidemiology of autoimmune diseases exhibits variability in incidence and prevalence rates, which becomes intricate when accounting for differences in age, gender, ethnicity, and additional demographic characteristics. The pathogenesis of autoimmune diseases involves a multitude of factors: 1. Microbiological Factors: Infections by bacteria and viruses are implicated in triggering autoimmune responses. 2. Environmental Influences: Exposure to chemicals, prolonged ultraviolet radiation, and lifestyle factors such as smoking and alcohol consumption may precipitate or intensify autoimmune reactions. 3. Genetic Predispositions: Certain human leukocyte antigen (HLA) alleles are significantly associated with the susceptibility to develop autoimmune diseases. 4. Hormonal effects: Fluctuations in hormone levels, gender and the use of certain medications, play a crucial role in disease onset, with women being disproportionately affected by specific autoimmune conditions (**Figure 1**). SLE is noted to have a prevalence ranging from 20 to 150 cases per 100,000 individuals, with a consistent increase in both incidence and prevalence across geographic regions (20). MS is typically diagnosed within the age range of 20 to 50 years (21). RA can affect individuals of any age, with peak incidence occurring between 50 and 59 years of age (22). SLE, MS, and RA are among the most prevalent and significant autoimmune diseases affecting patients, making them a central focus of research in the field of autoimmune disorders. This part will mainly provide a comprehensive review of these three diseases.

### 2.1 Systemic lupus erythematosus

SLE is a severe autoimmune disease characterized by a loss of immune tolerance, culminating in the immune system’s attack on the body’s healthy cells and tissues. This leads to immune complex-mediated inflammation across various organs, including the heart, kidneys, and skin (23). The mortality rate of patients with SLE is approximately 2–3 times higher than that of the general population, with geographical variations in prevalence and a female-to-male ratio of about 9:1 (24), affecting certain ethnic groups disproportionately, such as African Americans, American Indians, and Asians (25). The primary factors of mortality in SLE patients include renal disease, cardiovascular disease, and infections. Despite the approval of three novel treatments for SLE and lupus nephritis in recent years, the therapeutic management for the majority of patients has seen minimal evolution since the last century, with long-term outcomes characterized by high morbidity and mortality (26).

**Figure 2:**
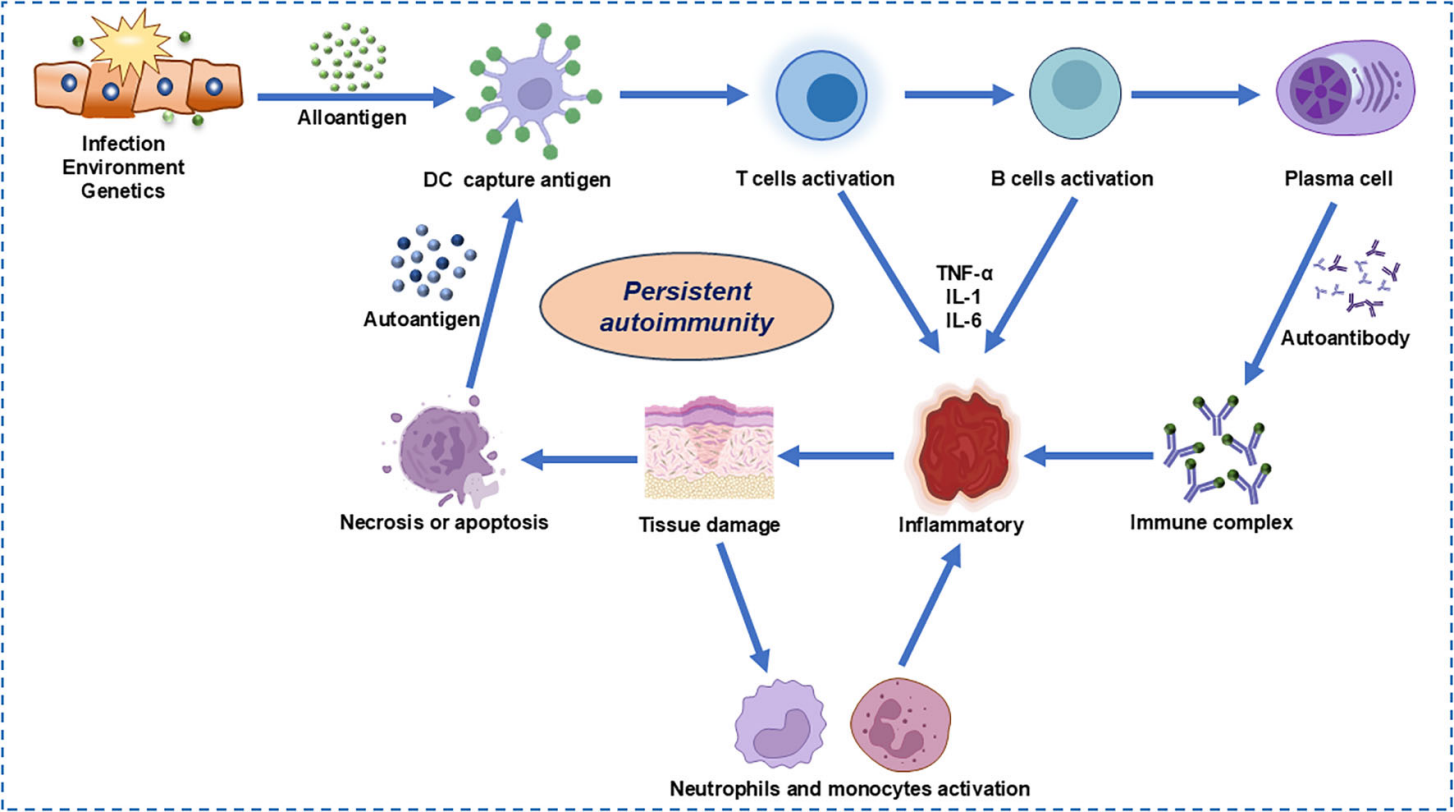
Cellular and molecular pathogenesis in autoimmune diseases.

The complex immunopathogenesis of SLE encompasses a multitude of factors including genetics, environment, hormonal influences, epigenetics, and immune modulation, all of which can impact the immune system either individually or in concert (24). A key aspect of this complexity is the impaired clearance of apoptotic cells, which leads to the accumulation of cellular debris (**Figure 2**). This debris can activate normally dormant autoreactive lymphocytes, potentially evading self-tolerance under conditions of recurrent or chronic stimulation (27). Furthermore, neutrophils, particularly low-density granulocytes, may exacerbate the intricate interplay between innate and adaptive immune responses. Through increasing the production of pro-inflammatory cytokines and forming neutrophil extracellular traps that contain immunostimulatory proteins and self-antigens, such as double-stranded DNA, the inflammation is perpetuated (28). In addition to these cellular mechanisms, the IFN-1 signaling pathway, which mimics a sustained antiviral response, is associated with lupus susceptibility and may amplify the autoimmune reaction. This amplification can be manifested by the enhancement of autoreactive humoral activity. Genome-wide association studies (GWAS) have identified at least 132 lupus susceptibility loci (29). The functional significance of these variants and their potential role in lupus expression remain largely unknown. Additionally, sex hormones and environmental influences may contribute to immune system dysregulation in genetically predisposed individuals (5). Current therapeutic approaches include glucocorticoids, immunosuppressants, and belimumab, which often fail to achieve drug-free remission.

### 2.2 Multiple sclerosis

MS is the most prevalent chronic inflammatory disease of the central nervous system (CNS) and the most common nontraumatic disabling condition affecting young individuals, impacting over 2 million people worldwide. It affects females at a ratio of approximately 3:1 and remains incurable. The majority of patients are diagnosed between the ages of 20 and 40, although children and the elderly can also be diagnosed with MS (30). Typical presenting syndromes of MS include, but are not limited to, monocular vision loss due to optic neuritis, limb weakness or sensory loss due to transverse myelitis, diplopia resulting from brainstem dysfunction, or ataxia caused by cerebellar lesions (31). MS can manifest as an inflammatory disorder characterized by episodes of neurological symptoms followed by partial or complete remission (relapsing-remitting MS, RRMS, in 85% of cases), or as a progressive disease (primary progressive MS, PPMS). Over time, RRMS may evolve into a progressive phase of the disease known as secondary progressive MS (SPMS) (32).

It remains unclear whether MS is attributable to a single cause or a combination of factors, as few specific triggers have been identified. Epstein-Barr virus (EBV), ultraviolet B (UVB) radiation, cigarette smoking, and vitamin D, in conjunction with an individual’s genetic background, all play significant roles in the development of MS. The prevalence of MS increases with latitude, a gradient closely related to UVB exposure, which stimulates the production of vitamin D in the skin. Low levels of vitamin D, reduced dietary intake of vitamin D, decreased outdoor activity, and increased susceptibility to MS associated with genetic polymorphisms linked to low vitamin D levels all correlate with the pathogenesis of MS (33). Furthermore, genetic factors influence susceptibility to MS, as approximately one-eighth of patients have a family history of the disease (34). GWAS conducted on samples collected from thousands of MS patients and matched controls have identified over 200 genetic variants that elevate the risk of developing MS, with the most significant association being linked to the HLA-DRB1*1501 haplotype (32).

### 2.3 Rheumatoid arthritis

RA is an organ-specific autoimmune disease primarily characterized by symmetric peripheral polyarthritis. It may also affect other organs, including the lungs, heart, blood vessels, skin, and eyes (35). The patients typically present with symmetric polyarthritis of the hands and/or feet, which can progress to joint destruction if left untreated (36). In individuals diagnosed with RA, there is often an overlap with other diseases or comorbidities, including cardiovascular disease, chronic lung disease, and periodontitis (37). RA affects approximately 0.5% to 1% of the global population, with a higher prevalence in regions farther from the equator, and the prevalence in women is two to three times greater than that in men. RA is classified as an autoimmune disease due to the presence of autoantibodies, such as rheumatoid factor (RF) and anticitrullinated protein antibodies (ACPAs), in most cases (38).

The etiology of RA remains unknown; however, certain risk factors are associated with an increased likelihood of developing the condition. These factors include the family history of RA or other autoimmune diseases, smoking, poor dental health, and viral infections. Several infectious agents are recognized as potential pathogens or promoters of RA, including EBV, retroviruses, bacterial superantigens, and mycoplasmas, as well as specific microorganisms such as *Porphyromonas gingivalis* and *Prevotella intestinalis*. The pathogenesis of RA is complex, with genetic susceptibility playing a crucial role. First-degree relatives of individuals with RA take a risk of developing the disease that is 2 to 5 times greater than that of the general population (39). GWAS conducted on a large scale have identified more than 100 loci associated with RA (40), including TNIP2, WISP1 and TNFRSF11A (41), and these alleles increase the risk of the disease and dramatically affect immune pathways. The most relevant alleles include the “common epitope” (SE) on the *HLA-DRB1* locus and protein tyrosine phosphatase (PTPN22) (42).

GWAS have unveiled shared genetic variations across multiple diseases, providing crucial clues for understanding the genetic foundations of autoimmune diseases conditions. By GWAS analysis, researchers have identified some common genetic markers for SLE, MS, and RA. Variant in the HLA region is particularly prominent in these diseases; as a key component of the human leukocyte antigen system, the polymorphisms of HLA genes are closely associated with susceptibility to autoimmune diseases (41). Specifically, the presence of HLA-DRB1*04:*01* and HLA-DQB1*03:*01* alleles significantly increases the risk of RA in individuals and promotes the development of an autoimmune response by affecting autoantigen presentation and T-cell responses. Additionally, specific variations in the PTPN22 gene, which is involved in the regulation of T-cell signaling, have been shown to be associated with an increased risk of various autoimmune diseases, including SLE and RA (29, 43). STAT4 plays a key role in Th1, Th17, and Tregs, and the single-nucleotide polymorphisms (SNP) rs7574865 for STAT4 is associated with increased risk of SLE and RA (44). The TNFSF13B gene, which is involved in the survival and differentiation of B cells, has variations that are associated with an increased risk of SLE (45). The IRF5 gene plays a role in the immune response, and several SNPs (rs4728142, rs2004640, rs1744583) and small insertions/deletions in the IRF5 gene or regulatory regions have been validated to independently cause SLE, MS and RA by altering gene expression, splicing, and RNA stability (46) These findings indicate that although SLE, MS, and RA have different clinical manifestations, there is a genetic overlap, which may reflect common mechanisms in their immune regulatory pathways. The outcomes of GWAS are of significant importance for elucidating the complex genetic structure of autoimmune diseases, advancing the development of precision medicine, and developing new therapeutic approaches.

Autoimmune diseases exhibit a pronounced sexual dimorphism, with a higher incidence rate among females than males for numerous conditions. This gender bias may stem from a convergence of factors, including sex chromosome disparities, hormonal influences, the activity of the Xist ribonucleoprotein complex, gender-based immune cell variations, environmental exposures, and genetic predispositions. The presence of two X chromosomes in females could contribute to distinct immunomodulatory gene expression patterns, as the X chromosome is enriched with such genes, and variations in X chromosome number may influence susceptibility to autoimmune diseases (47). Men with Klinefelter syndrome (XXY) have an extra X chromosome, which causes them to experience significant impairments in fetal germ cell (FGCs) development, such as stagnation of FGCs at an early age, abnormal dosage of X-linked genes, aberrant interactions of Sertoli cells with FGCs, and inhibition of the TGF-β signaling pathway to improve FGCs differentiation (48). Disturbances in these molecular mechanisms not only affect their reproductive health, but are also associated with an increased risk of autoimmune diseases. Patients with KS may have abnormalities in their immune system, including abnormalities in the number and function of immune cells and a chronic inflammatory state, which together may put them at higher risk for autoimmune diseases (49). Recent studies have further revealed how abnormalities in gene dosage on the X chromosome can affect the risk of autoimmune diseases, particularly in patients with KS. The extra X chromosome leads to increased expression of X-linked genes such as *Tlr7*, a single-stranded RNA sensor associated with the pathogenesis of autoimmune diseases. Research has found that in KS patients, the X chromosome does not undergo inactivation in FGCs, resulting in an excess of X-linked gene dosage, which may directly impact the function of immune cells and the regulation of autoimmune responses. Additionally, a 3.2MB region of the X chromosome has been translocated to the Y chromosome, and this previously unreported X-Y translocation may influence the risk of autoimmune diseases in KS patients (50). These findings underscore the importance of X chromosome dosage control for maintaining normal immune function and provide a new perspective on how gender affects the pathogenesis of autoimmune diseases, highlighting the significance of gender differences in autoimmune disease research. Additionally, fluctuations in sex hormones like estrogen and progesterone may perturb immune cell development and activity, disrupting immune tolerance. The Xist RNP complex, responsible for X chromosome silencing in females, also forms molecular complexes implicated in autoimmune pathogenesis (51). Furthermore, environmental factors such as infections and ultraviolet radiation may impact the immune system in a gender-specific manner, and certain genetic variants are more prevalent in women, potentially increasing their risk for autoimmune diseases. The interplay among these factors likely drives the higher incidence of autoimmune diseases in women, highlighting the complexity of sex-based differences in disease susceptibility.

## 3 Emerging cell-based therapies for autoimmune diseases

**Figure 3:**
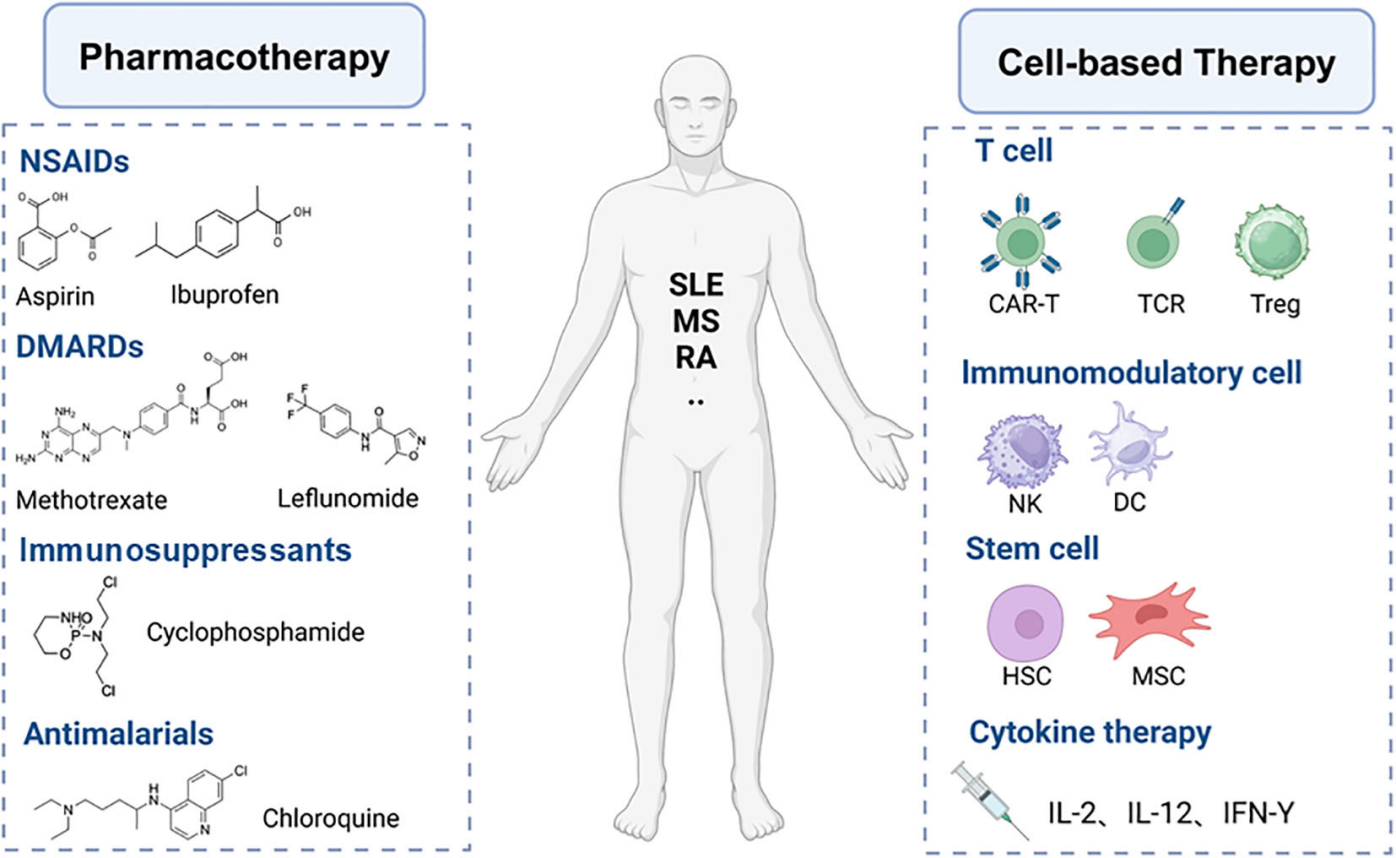
Pharmacotherapy and cell-based therapy for autoimmune diseases.

The underlying causative factors of autoimmune diseases remain largely unknown; however, two main reasons contribute to the failure of immune tolerance (1) the presence of autoantibodies and (2) the presence of disease-associated autoreactive lymphocytes (52). The complexity and heterogeneity of the mechanisms underlying immune dysregulation in autoimmune diseases present numerous challenges to develop novel therapeutic strategies that offer specific, long-lasting efficacy with minimal side effects (53). The treatment of autoimmune diseases currently relies predominantly on pharmacotherapy, which includes hormones, immunosuppressants, immunomodulators, and anti-inflammatory drugs (**Figure 3**). The mechanisms of these medications in treating autoimmune diseases are diverse, encompassing the suppression of inflammatory responses, modulation of immune system functions, and reduction of the number or activity of autoreactive cells. For instance, glucocorticoids broadly inhibit immune responses, while immunosuppressants such as cyclophosphamide and methotrexate reduce the number of immune cells by inhibiting DNA synthesis and cellular proliferation (54). Biologic DMARDs, such as infliximab and anifrolumab, are monoclonal antibodies targeting specific inflammatory mediators or immune cells, allowing for a more precise regulation of immune responses (55). JAK inhibitors reduce the signaling of inflammatory cytokines by suppressing the activity of JAK kinases (56). Natural compounds like curcumin and resveratrol also show therapeutic potential due to their broad targets, favorable safety profiles, and potential immunomodulatory effects (57). Although existing treatment modalities can control the disease to a certain extent, the complexity of autoimmune diseases often necessitates personalized treatment and the combined use of multiple drugs to achieve optimal efficacy. Moreover, pharmacological treatments often come with limitations and side effects, such as the severe side effects associated with long-term dependence on glucocorticoids and immunosuppressants, as well as the high costs and treatment limitations of biologic agents (58). Despite the introduction of improved therapies for autoimmune conditions in recent decades, further advancements are necessary. Ongoing research of cellular therapy strategies holds the potential to transform the traditional treatment paradigm for these diseases, offering the possibility of achieving sustained, targeted immune modulation while preserving essential protective immune functions.

Cell-based therapies (**Figure 3**) offer significant advantages over antibodies and cytokines in restoring immune tolerance due to the persistence and efficacy of these therapeutic cells *in vivo (* 59). Such treatment approaches include the expansion of IL-10-expressing TR1 cells (60) and the engineering of Treg cells to express specific TCRs or CARs (61, 62), which modulate immune responses and contribute to the suppression of excessive autoimmune reactions. Moreover, MSCs provide a novel strategy for the treatment of autoimmune diseases by secreting anti-inflammatory factors and promoting tissue repair. HSCs, as multipotent stem cells capable of differentiating into various immune cells, offer a new perspective on immune modulation. CAR-T cells have shown promise in the treatment of autoimmune diseases by eliminating pathologically activated immune cells or re-establishing immune tolerance in affected organs (11). CAAR-T cells and CAR-NK cells utilize their specificity and enhanced targeting capabilities to provide innovative avenues for the treatment of autoimmune diseases. The innovative application of these cellular therapy strategies not only broadens our understanding of the treatment of autoimmune diseases but also provides a diverse array of options for future clinical therapies.

### 3.1 Treg cells

Tregs constitute 5-7% of CD4+ T cells and develop both directly in the thymus (thymus-derived Tregs, tTregs) and in peripheral tissues (peripherally-derived Tregs, pTregs). They possess the ability to suppress immune responses and promote tissue repair, ensuring that the immune system maintains a proper balance between its responses to foreign antigens and self-antigens (63). Treg cells are located in inflamed tissues and localized secondary lymphoid organs, where they exert their immunosuppressive functions. The number and functionality of Treg cells are altered in autoimmune diseases, resulting in defects in immune tolerance, abnormalities in immunoregulation, increased inflammation, and the proliferation of autoimmune cells. Treg cells confer immune tolerance through various mechanisms, including the secretion of anti-inflammatory soluble mediators such as interleukin-10 (IL-10), transforming growth factor-beta (TGFβ), and interleukin-35 (IL-35). They also achieve this by depleting interleukin-2 (IL-2) and expressing cytotoxic T lymphocyte antigen 4 (CTLA-4), CD39, CD73, and other inhibitory cell-surface receptors (64). Furthermore, Tregs regulate the direct or indirect targeting of T cells by APCs. For instance, the binding of CTLA-4 to CD80/CD86 on APCs induces the enzyme indoleamine-2,3-dioxygenase (IDO) (65). Additionally, Treg binding to APCs can result in the removal of cell surface molecules (a process akin to phagocytosis), thereby altering co-stimulation and antigen presentation (66).

Several therapeutic strategies based on the function of Treg cells have been proposed to restore tolerance in affected tissues. Transcription factors in Treg cells play a pivotal role in maintaining immune tolerance, preventing autoimmunity, modulating immune responses, promoting immune homeostasis, and supporting transplantation tolerance. Forkhead box P3 (Foxp3) plays a crucial role in the differentiation, development, and functional stabilization of Tregs. Fluctuations in Foxp3 protein levels can alter the stability of Treg function. Such variations are associated with a spectrum of significant immune-related disorders in humans. These include infectious diseases, autoimmune diseases, allergic diseases, tumorigenesis and metastasis, as well as transplant rejection. The Foxp3 gene has thirteen mutations, including five within the forkhead domain, one causing a frameshift due to a nucleotide deletion, and another disrupting the leucine zipper domain through base deletion. Such mutations may destabilize the mRNA, resulting in reduced levels of normal Foxp3 protein in T cells. This can lead to immune-mediated disorders such as diabetes, lymphadenopathy, and cytokine storms *in vivo (* 67). The therapeutic potential of Foxp3+ Tregs has been demonstrated in various preclinical models such as graft-versus-host disease (GVHD) (68), type 1 diabetes mellitus (T1D) (69), systemic lupus erythematosus (70), inflammatory bowel disease (71) and multiple sclerosis (72). Currently, over 50 clinical trials are either underway or have been completed to assess the safety and efficacy of Treg cell therapy for the treatment of conditions such as renal or liver transplantation, atypical forms of pemphigus, systemic lupus erythematosus, inflammatory bowel disease, autoimmune hepatitis, allergies, and asthma (73).

Treg cells play a crucial role in suppressing immune responses and maintaining immune homeostasis. The infusion of polyclonal Treg cells has demonstrated a favorable safety profile. The efficacy of this approach has been established in patients with type 1 diabetes mellitus and those with GvHD, with additional clinical trials currently underway for patients with Crohn’s disease and aspergillosis. IL-2 is a vital factor in promoting Treg cell expansion by regulating the STAT5 signaling pathway and enhancing Foxp3 expression, which is essential for Treg cell function and proliferation (74). Consequently, IL-2 analogs, such as IL-2/antibody complexes and IL-2 mutant proteins, represent promising therapeutic strategies for treating autoimmune diseases. In August 2013, a phase I/IIa study (NCT02084238) led by Peking University People’s Hospital showed that the administration of low-dose IL-2 has been shown to stimulate Treg cell expansion of patients with various autoimmune conditions involving (75). Amgen’s AMG-592, an IL-2 mutein with increased regulatory T cell selectivity, is in phase I/II trials in systemic lupus erythematosus and graft-versus-host disease, which also showed enhanced Treg cell expansion after low-dose IL-2 infusion in patients with SLE and type 1 diabetes (76) However, the limitations of IL-2 therapy include its short half-life, the necessity for repeated injections, and the potential for anti-drug immune responses. To address these challenges, Treg cells have been engineered, including those with transgenic TCRs or CARs, which have shown improved efficacy in targeting specific antigens and suppressing effector responses compared to polyclonal Treg cell infusion.

### 3.2 MSCs

MSCs are pluripotent progenitor cells capable of supporting hematopoiesis and differentiating into various mesodermal cell lineages, such as osteoblasts, chondrocytes, and adipocytes (77). They possess significant repair potential through self-renewal and differentiation and are increasingly recognized for their ability to modulate the immune response, exhibiting immunomodulatory properties. MSCs are believed to play a crucial role in the formation of memory T-cell and B-cell survival niches in the bone marrow and interact effectively with a variety of myeloid and innate leukocytes, including dendritic cells, monocytes, and macrophages (78). This interaction modulates immune memory size, stability, and plasticity. Initially, MSCs were found to inhibit mitogen-induced T-cell proliferation *in vitro* and evade immune surveillance *in vivo*. Subsequent studies have demonstrated that MSCs can modulate immune responses during chronic inflammation by regulating cell recruitment, function, and fate within the innate and adaptive immune systems (79). Following extensive *in vitro* and *in vivo* preclinical trials, both autologous and allogeneic MSCs have been utilized in the treatment of various immune-mediated diseases, including graft-versus-host disease, Crohn’s disease, multiple sclerosis, refractory systemic lupus erythematosus, and systemic sclerosis.

It is now established that autoreactive T lymphocytes, particularly CD4/helper T cells, play a crucial role in the development of autoimmune diseases. A significant finding across most autoimmune conditions is the imbalance between the effector T-cell subsets Th1/Th17 and Tregs, which produce the immunomodulatory cytokine IL-10. This imbalance ultimately leads to inflammation and damage in target tissues. Given that the immunomodulatory effects of MSCs have been shown to be most effective on CD4+ T cells, it is not surprising that MSCs have been utilized in the highest number of clinical trials aimed at treating autoimmune diseases. Currently, over 25% of MSC-related trials focus on autoimmune conditions; these 129 trials include 34 for inflammatory bowel disease (IBD), 25 for multiple sclerosis, 18 for type 1 diabetes, 16 for systemic lupus erythematosus, 12 for RA, 9 for psoriasis, and 15 for other autoimmune diseases. The majority of these trials are in the early phases, with 29 in phase I, 15 in phase II, and 73 in combined phases I/II. Additionally, there are seven ongoing trials assessing efficacy, including three in combined phases II/III, three in phase III, and one in phase IV. Furthermore, five trials have not yet been assigned a phase (80).

In a comprehensive analysis by Zeng et al (81), the therapeutic efficacy and safety of MSC transplantation were evaluated across five autoimmune diseases, encompassing 18 randomized controlled trials. Among patients with RA, a trio of trials by Yang et al (82) indicated that MSC treatment led to reduced disease activity, enhanced standing time over 50% alleviation in knee pain, and decreased medication reliance, with benefits persisting up to 12 months in the majority of cases. In the context of SLE, four randomized controlled clinical trials assessed parameters such as urinary protein levels, serum complement C3, and adverse events following MSC intervention. These trials revealed lower urinary protein levels and alongside elevated serum C3 levels in the treatment group compared to the control group, with no significant adverse events reported across all studies (83). Six trials concerning MS demonstrated that patients who received MSC therapy exhibited superior progression-free survival (PFS), a reduced total number of episodes, and without encountering any severe adverse events (84).

### 3.3 HSCs

The core pathological characteristic of autoimmune diseases is the aberrant attack of the immune system on self-tissues, leading to chronic inflammation and tissue damage. This conundrum of immune dysregulation has prompted the medical community to explore alternative cellular therapies, aiming to reset or rebalance the immune system and restore tolerance to self-antigens. In this field, HSCs have emerged as ideal candidates for the treatment of autoimmune diseases due to their unique immunomodulatory properties (85, 86). HSCs are capable of repopulating the immune system, reducing the number of autoreactive lymphocytes, and by secreting cytokines such as TGF-β and IL-10, they modulate the immune microenvironment, fostering the formation of an anti-inflammatory milieu (87). Furthermore, HSCs can differentiate into Tregs and other immunomodulatory cells, which play a crucial role in suppressing autoimmune responses.

Based on these mechanisms, hematopoietic stem cell transplantation (HSCT) has been established as a standard therapeutic approach for malignant hematological disorders, other oncological conditions, and severe immunodeficiencies (88). For patients with autoimmune diseases who have an inadequate response to conventional treatments, HSCT following high-dose chemotherapy is increasingly being utilized in clinical practice. The therapeutic principle of HSCT involves initial lympholysis to reduce the immune memory repertoire, including autoreactive clones, followed by a profound immune renewal through the regeneration of hematopoiesis and the immune system (10). This process not only offers patients the opportunity to rebuild their immune system but also provides the possibility for long-term remission of autoimmune diseases. In 2012, it was estimated that approximately 3,000 patients with autoimmune diseases worldwide received HSCT treatment (89). As clinical research progresses, the application prospects of HSCT in the treatment of autoimmune diseases are expected to expand further.

HSCT has demonstrated significant potential in addressing severe, refractory SLE. Multiple research outcomes have indicated that HSCT can markedly improve the disease prognosis for SLE patients while reducing their reliance on long-term immunosuppressive drug therapy. Since its initial application in the treatment of SLE in 1997, over 300 cases of autologous hematopoietic stem cell transplantation have been reported globally (90). Furthermore, autologous HSCT has shown positive effects in patients with aggressive MS, not only facilitating the recovery of neurological functions but also successfully halting the disease’s progression. For patients with RA unresponsive to conventional therapies, HSCT treatment has achieved long-term remission of the condition. In 1996, the first case of autologous HSCT for a patient with rheumatic autoimmune disease was reported (91); the patient, who faced rejection for lung transplantation due to connective tissue disease and severe pulmonary arterial hypertension, ultimately benefited from HSCT. To date, more than 3,000 HSCT procedures have been conducted worldwide for patients with severe rheumatic and non-rheumatic autoimmune diseases (87). These data underscore the efficacy and feasibility of HSCT in treating certain autoimmune diseases, offering new directions for future therapeutic approaches.

HSCT offers a potentially transformative approach for the treatment of autoimmune diseases but still faces challenges such as GVHD, infection risks, donor matching difficulties, conditioning regimen toxicity, graft failure, and long-term complications. However, with the advent of non-myeloablative conditioning regimens, improved HLA typing techniques, gene-editing technologies, the development of new anti-rejection drugs, the expansion of indications, and the emergence of “off-the-shelf” stem cell products, the prospects for HSCT are continuously improving. This evolution provides patients with safer, more effective, and more accessible treatment options, expected to enhance long-term survival rates and quality of life.

### 3.4 CAR-T cells

With the inherent ability of T cells to infiltrate tissues, their high-affinity binding to specific targets, and their antitumor effector functions, CAR-T cell therapy is predicated on the precise targeting of tumor antigens, resulting in the lysis and destruction of tumor cells. CAR-T cells offer several advantages over monoclonal antibodies, as T cells are long-lived, capable of proliferation, can be transported to lymphoid tissues or target organs, and can develop memory populations that help prevent the recurrence of disease-causing lymphocytes (92). Zelig Eshhar first demonstrated a CAR prototype in 1982, which shares many conceptual similarities with the CAR structures used today (93). The cytoplasmic portion of the CAR includes signaling domains (the CD3ζ chain of the T-cell receptor) and costimulatory domains (such as 4-1BB or CD28) to ensure the proper expansion and activation of CAR-T cells, as well as the proliferation of target cells. The CD19 antigen, expressed by B-cell-derived malignancies such as lymphomas and leukemias, was the first clinically applicable target for cancer immunotherapy using autologous CAR-T cells (94).

CAR-T cells demonstrate significant potential in the treatment of autoimmune diseases due to their ability to eliminate pathologically activated immune cells and to reestablish immune tolerance in organs affected by immune dysregulation. The number of studies investigating the use of CARs for treating autoimmune diseases is rapidly increasing (**Table 1**), with a particular focus on CAR-T cells and T cells (95). In 2021, CAR-T cells targeting CD19 were first utilized to treat a 20-year-old woman with severe treatment-refractory SLE (96). This approach confirmed the feasibility of producing CAR-T cells from patients with autoimmune diseases, and patient compliance with CAR-T cell infusions like this is high without any serious toxic reactions. This marked the first CAR-T cell-based treatment for an autoimmune disease, and additional clinical trials using anti-CD19/BCMA CAR-T cells or anti-CD19 CAR-T cells for relapsed/refractory SLE are currently ongoing (97). Using CD19-targeted CAR-T cell therapy, some researchers successfully treated a patient with anti-synthetase antibody syndrome, who fully recovered from the autoimmune disease without the need for immunosuppressive medications six months after CAR-T cell therapy (98). Over the past two years, CAR-T cells have also been employed in preclinical trials for MS, type 1 diabetes mellitus, inflammatory bowel disease, SLE, and pemphigus vulgaris (PV) suggesting new hope for therapeutic options in autoimmune diseases (99). In RA, one study designed CAR-T cells to eliminate specific autoreactive B cells by citrullinating antigenic epitopes, resulting in the lysis of B-cell subsets (100). Additionally, another study constructed CD8 T cells expressing the *HLA-DR1* CAR, which led to a reduced CD4 T cell response and inhibition of autoantibody production, indicating the potential for a highly specific therapeutic approach in the treatment of autoimmune diseases (101).

**Table 1:** The status of CAR-based therapies in autoimmune diseases (until 05/2024).

Target antigen	Cell type	Type of disease	Clinical Trials	Research status
CD19	CAR T	Systemic lupus erythematosus	NCT03030976	Phase 1
CD19, BCMA	CAR T	Systemic lupus erythematosus	NCT05030779	Phase 1
BCMA	mRNA CAR T	Generalized myasthenia gravis	NCT04146051	Phase 2
CD19/BCMA/CD138/BAFF-R	CAR T	Autoimmune diseases	NCT05459870	Phase 2
PD-1	CAR T	Primary biliary cholangitis	——	Preclinical study
BCMA/CD19	CAR T	Relapsed/Refractory systemic lupus erythematosus	NCT05474885	Phase 1
CD7	CAR T	Crohn diseases; Ulcerative colitis	NCT05239702	Phase 1
CD19/CD20	CAR T	Neuromyelitis optica spectrum disorder	NCT03605238	Phase 1
BCMA	CAR T	Neuromyelitis optica spectrum disorder	NCT04561557	Phase 1/2
HLA-A2 antigens	CAR Treg	HLA-A2 mismatched liver transplantation	NCT05234190	Phase 1/2
MuSK	CAAR T	MuSK myasthenia gravis	NCT05451212	Phase 1
DSG3	CAAR T	Mucosal-dominant pemphigus vulgaris	NCT04422912	Phase 1

The expression of CAR in Tregs represents a promising strategy to enhance the efficacy and specificity of Treg therapy. Researchers have developed CAR-Tregs that target carcinoembryonic antigen (CEA) for the treatment of ulcerative colitis (UC). These anti-CEA CAR-Tregs specifically inhibited colitis symptoms in various experimental UC models using the colons of CEA transgenic mice (102). In a landmark study, researchers engineered CAR-Tregs against 2,4,6-trinitrobenzene sulfonic acid (TNBS) in a mouse model of colitis (103). CAR-Tregs secrete inhibitory factors, proliferate, and ameliorate disease symptoms in an antigen-specific manner. Similar findings have been reported in mouse models of multiple sclerosis and transplant rejection (104–106). CAR-Tregs demonstrate therapeutic efficacy at doses comparable to those of non-engineered Tregs, indicating that CAR expression not only enhances the efficacy of Treg therapy but also increases its specificity. Overall, these studies provide a robust theoretical foundation for clinical trials of CAR-Treg therapy.

Transient mRNA-based CAR-T cell therapy has emerged as a promising approach for the treatment of autoimmune diseases. Conventional CAR-T cell therapies utilize lentiviral or γ-retroviral vectors to introduce permanent genetic modifications to T cells, which carry risks of genotoxicity and regulatory challenges. Additionally, CAR-T cells may persist for a lifetime (107). Given that the safety standards for autoimmune trials are exceptionally high—often exceeding those for cancer trials—mRNA-based CAR-T cell therapies present an alternative strategy for delivering CAR-encoded mRNAs to T cells without permanently altering their genomes. This method allows for transient, time-limited expression of CARs, providing a controlled and reversible therapeutic approach. In a phase 1b/2a clinical study, mRNA-based CAR-T cell therapy demonstrated the potential for more durable symptomatic relief of myasthenia gravis and was well tolerated, with no significant adverse effects reported in patients (108). However, the potential immune response to CAR-T cells, the need for precise dosing and treatment timing, and the requirement for Good Manufacturing Practice (GMP)-compliant drug manufacturing have yet to be fully addressed in this rCAR-T cell study.

In the context of malignant diseases, the risk of immune escape, along with tumor recurrence and toxic effects, represents one of the most significant limitations of CAR-T cell therapy Cytokine release syndrome is particularly concerning as a toxic effect of CAR-T cell therapy; mild cases may present with symptoms such as fever, headache, arthralgia, and myalgia, but severe cases can lead to hypotension and even cytokine storm. Another common side effect is immune effector cell-associated neurotoxicity syndrome, which can manifest as fine motor deficits, resulting in writing difficulties and altered speech. Additional symptoms associated with immune effector cell-associated neurotoxicity syndrome include headache, confusion, seizures, and behavioral changes. Therefore, further basic and preclinical studies are essential to evaluate CAR-T cell-based therapies.

### 3.5 CAAR-T cells

Significant success has been reported in the use of anti-CD19/anti-CD19 BCMA CAR-T cells for the treatment of cancer and SLE. However, these approaches result in the depletion of all B cells in the body (109). Consequently, B cells may be absent for weeks to months, leaving patients immunocompromised during this period. To address this widespread B-cell depletion, researchers have developed CAARs that specifically and precisely target pathogenic B-cell subsets. Therapeutic T cells are genetically modified with chimeric receptors that incorporate target antigens for autoantibodies as extracellular structural domains. Unlike CAR-T cells, which express molecular receptors found on pathological cells, CAAR-T cells express extracellular autoantigens recognized by the B-cell receptor (BCR) (110). This extracellular structural domain is linked to the transmembrane structural domain, the costimulatory structural domain, and the activation structural domain, similar to the intracellular components of CARs.

Autoantigen recognition by autoreactive B cells leads Autoantigen recognition by autoreactive B cells leads to the activation of CAAR-T cells and the specific lysis of pathogenic B cells. Findings from a preclinical mouse model of atypical pemphigus suggest that CAAR-T cells expressing Desmoglein 3 may be effective in treating the rare skin disease known as pemphigus vulgaris (99). In a recent study, researchers developed a novel therapy for the most prevalent form of autoimmune encephalitis, specifically NMDA receptor encephalitis (110). Programmed CAAR-T cells are designed to recognize and eliminate anti-NMDA receptor antibody-producing B cells with high precision, and this innovative approach demonstrated its efficacy in a mouse model. Muscle-specific tyrosine kinase myasthenia gravis (MuSK MG) is an autoimmune disorder characterized by life-threatening muscle weakness due to the presence of anti-MuSK autoantibodies, which disrupt neuromuscular junction signaling. Researchers engineered T cells expressing the MuSK chimeric autoantibody receptor with a CD137-CD3ζ signaling domain (MuSK-CAART) to specifically target B cells that produce anti-MuSK autoantibodies (111). In an experimental autoimmune MG mouse model, MuSK-CAART effectively reduced anti-MuSK IgG levels without affecting overall B-cell counts or total IgG levels, indicating the selective depletion of MuSK-specific B cells. While CAAR-T cell approaches have shown promise in treating various autoimmune disorders, their development is limited to diseases caused by single autoantibody-producing monoclonal B-cell clones.

### 3.6 CAR-NK cells

A total of six CAR-T cell therapies have been approved to date, demonstrating significant promise primarily for the treatment of hematological malignancies. However, this approach is effective only in a subset of patients and is associated with considerable side effects, such as cytokine release syndrome and neurotoxicity, which further limits its broader application in cancer treatment (112). To address these limitations, researchers have turned their attention to NK cells, which are integral to the innate immune response—the body’s first line of defense against infections—and play crucial roles in antiviral, anticancer, and anti-aging processes. NK cells, along with T and B cells, form a major subset of lymphocytes capable of nonspecific, non-MHC-restricted direct tumor cell killing (97). NK cells are pivotal in cancer immunity as they target cancer cells that downregulate HLA class I molecules or express stress markers. Furthermore, NK cells can be genetically modified to express CARs and can be utilized without the need for recipient-matched human leukocyte antigens, thereby eliminating the necessity for patient-specific production of CAR products. One study demonstrated that lupus-like mice treated with CAR-NK cells exhibited improved splenomegaly and a reduction in the number of PD-1+CD4+ T cells (113). Additionally, a clinical phase 1/2 trial involving umbilical cord blood-derived NK cells expressing an anti-CD19 chimeric antigen receptor and IL-15 (cAR19/IL-15) was conducted with 37 patients suffering from CD19+ B-cell malignancies. The results indicated that none of the patients developed neurotoxicity or graft-versus-host disease, and only one patient experienced cytokine release syndrome (grade I) (114). These findings underscore the feasibility of utilizing CAR-NK cells for cancer treatment.

According to previous studies, there exists an innate lymphocyte population derived from NK cells known as tissue-resident memory natural killer (NKRM) cells. These cells play a crucial role in regulating immune responses within tissues by preventing the immune system from mistakenly attacking its own tissues or organs, thereby helping to avert autoimmunity (115). Researchers have preliminarily demonstrated that NKRM cells exhibit distinct immune functions compared to traditional memory cells; they can eliminate CD4+ T cells, reduce autoimmunity in a TRAIL-mediated manner, and may hold significant potential for research into the treatment of SjD (115). The results of this study revealed that four patients with severe disease achieved deep remission after treatment with these cells, transitioning from “severe” to “mild” disease, with corresponding durability. Additionally, two patients with severe disease attained deep remission after just 15 days of treatment, with SLEDAI-2K disease scores decreasing from 14 and 17 points (indicating severe disease) to 1 and 3 points (indicating mild disease), respectively. This indicates a highly positive effect of CAR-NK cell therapy. This study represents the first international clinical report on the use of CAR-NK cells for the treatment of SLE.

## 4 Other emerging therapies

Gene therapy has emerged as a promising approach for the treatment of autoimmune diseases, with the CRISPR-associated protein 9 (CRISPR-Cas9) system being one of the most prominent methods. The CRISPR gene-editing technology enables rapid and efficient generation of gene knockouts, modulation of endogenous gene expression, and replication of genomic alterations associated with cancer. The CRISPR-Cas9 technique has demonstrated potential in the therapeutic research of various autoimmune diseases, including RA, SLE, MS, type 1 diabetes and psoriasis (116). Xu et al. are exploring the use of CRISPR-Cas9 technology to modulate immune responses, including the knockout or modification of specific immune cells to reduce autoimmune reactions (117). For instance, by knocking out certain genes in T cells, the function of Tregs can be enhanced, thereby suppressing excessive immune responses. A research team, after constructing CAR-T cells targeting CD19, used the CRISPR-Cas9 gene-editing tool to knockout five genes in the CAR-T cells (HLA-A, HLA-B, CIITA, TRAC, and PD-1) to avoid the graft-versus-host disease caused by allogeneic T cells. This led to the development of a new generation of off-the-shelf CAR-T therapy (TyU19), which successfully treated one patient with refractory immune-mediated necrotizing myopathy (IMNM) and two patients with diffuse cutaneous systemic sclerosis (dcSSc) (118). During the 6-month follow-up after treatment, all three patients experienced profound symptom relief, significant improvement in disease clinical response index scores, and reversal of inflammation and organ fibrosis. The entire treatment process for the three patients was well-tolerated, with no observed CRS, GvHD, or immune effector cell-associated neurotoxicity syndrome (ICANS) commonly seen in cancer patients receiving CAR-T cell therapy. These findings highlight the potential of this novel treatment approach to provide safer and more effective therapeutic options for patients with autoimmune diseases. With continuous technological advancements and the conduct of clinical trials, it is anticipated that more applications of CRISPR-Cas9 will be developed in the future to improve the therapeutic outcomes for autoimmune diseases.

In the quest for alternative immunotherapies for autoimmune diseases, the Proteolysis-targeting chimera (PROTAC) technology has emerged as a promising new therapeutic strategy. This technique harnesses the ubiquitin-proteasome system (UPS) and small molecules to achieve the degradation of specific target proteins, offering a novel approach to treatment (119). Bruton’s tyrosine kinase (BTK), a crucial regulator of B cell development, proliferation, activation, and differentiation, has become a focal point for therapies targeting B cell malignancies and autoimmune diseases. The role of BTK in B cell-related conditions positions it as a key target for intervention (120). Although BTK inhibitors show potential in treating autoimmune diseases, the clinical outcomes have been mixed due to challenges in efficacy and safety. To address these issues, Liu et al. have developed a new generation of BTK-PROTAC degraders, such as L18I, which demonstrate significant efficacy in autoimmune disease mouse models (121). L18I effectively alleviates symptoms of lupus and diffuse alveolar hemorrhage (DAH) by reducing autoantibody production and mitigating inflammatory responses. Moreover, L18I exhibits high selectivity for proteins that Ibrutinib, a BTK inhibitor, struggles to target, such as ITK, EGFR, and HER2. This indicates that L18I can effectively degrade BTK in various tissues, making it a strong candidate for the treatment of autoimmune diseases. These findings underscore the potential of PROTAC technology in developing innovative therapeutic strategies for autoimmune diseases and highlight the importance of degraders targeting key signaling molecules like BTK in modulating immune responses and alleviating disease symptoms. With further research and optimization of these emerging therapies, we anticipate they will offer more effective and safer treatment options for patients with autoimmune diseases.

## 5 Outlook

Current strategies for the treatment of autoimmune diseases primarily involve the use of immunosuppressive drugs and biologically targeted therapies; however, none have yielded satisfactory clinical outcomes. The rapid advancement of cell-based therapies and synthetic immunology approaches has expanded their potential to treat human diseases. In this paper, we present a comprehensive overview of three prevalent autoimmune diseases and six research advancements in cellular therapy for these conditions.

Cellular therapy represents a novel approach for treating tumors and one FDA-approved engineered immune cell therapy, known as CAR-T cell therapy, has demonstrated success in treating specific B-cell malignancies. In recent years, the application of immune cell therapies has expanded to include the treatment of autoimmune diseases, yielding several promising results. However, despite the potential indicated by preclinical studies, no immune cell therapies have yet received approval for the treatment of autoimmune diseases, and only a limited number of approaches have progressed to phase 1 or phase 2 clinical trials.

As immunotherapy for autoimmune diseases is often not initiated until the disease has progressed significantly, resulting in tissue damage and inflammation, the identification and validation of biomarkers that can predict therapeutic response and monitor disease progression will enable a more precise cellular therapy strategy. The current approach of long-term immunosuppression in treating autoimmune diseases could be transformed by cellular therapy into a strategy that induces an immune reset without the need for ongoing treatment. A deeper understanding of the pathological mechanisms underlying autoimmune diseases, coupled with recent advances in cell manufacturing technologies, will facilitate the development of novel and potent therapies that fundamentally alter cell–cell interactions and improve clinical outcomes.

Cellular therapy, as an emerging treatment modality, offers new avenues for the management of autoimmune diseases, yet it also presents challenges related to safety, ethics, cost, and technical expertise. In terms of safety, vigilant monitoring is required for potential side effects such as CRS and neurotoxicity associated with CAR-T cell therapy, while the stability and durability of Tregs and MSCs therapies must also be assessed. The uncertainty surrounding long-term efficacy and disease recurrence rates necessitates long-term follow-up studies to evaluate the persistence of treatments and the potential for relapse. On the ethical front, since cellular therapy technologies are often in experimental stages, it is crucial to ensure that patients fully comprehend the risks of treatment, possible adverse reactions, and the uncertainty of therapeutic outcomes. Moreover, the high cost and complexity of cellular therapy preparation demand sophisticated technical skills, posing a challenge for many medical institutions and limiting the broad application of these therapies. Therefore, optimizing manufacturing processes, enhancing the stability of cell products, and fostering international collaboration to share data and harmonize clinical trial standards are essential for reducing costs, increasing accessibility, and advancing the field.

Autologous cell therapies, such as autologous CAR-T cell therapy, utilizes the patient’s own cells for modification to avoid rejection reactions. However, the preparation process is complex and time-consuming. Allogeneic cell therapies, which utilize native immune cells or cells derived from induced pluripotent stem cells (iPSCs), show broad application prospects due to the potential for mass production and cost-effectiveness. Allogeneic iPSC technology has shown tremendous potential in treating a variety of diseases, including neurological disorders, cardiovascular diseases, and autoimmune diseases. The world’s first off-the-shelf CAR-T therapy (TyU19) for the treatment of autoimmune diseases has demonstrated significant efficacy and safety in clinical trials (122). Concurrently, in the treatment of autoimmune diseases, Tregs derived from allogeneic iPSCs have also shown therapeutic potential. Professor Shin Kaneko and his team from Kyoto University, have successfully induced Treg-like cells from iPSCs-derived conventional helper T cells (Tconvs) and confirmed their ability to suppress xenogeneic GvHD (123). Both autologous and allogeneic cell therapies have shown their respective advantages and challenges in the treatment of autoimmune diseases. As technology continues to advance and clinical trials progress, the future application prospects of iPSC in the field of cell therapy will be even broader.

In conclusion, cellular therapy demonstrates significant potential and promise in the treatment of autoimmune diseases, but they still require further research and validation in terms of efficacy, safety and cost-effectiveness. Future research aims to enhance the efficacy of immunomodulatory treatments while avoiding adverse reactions and to develop personalized immunocyte therapy strategies using patients’ specific genetic and immunological information to improve treatment success rates. Additionally, exploring the integration of cell therapy with traditional drug treatments, physical therapies, and other treatment modalities to form comprehensive treatment strategies is an important direction for enhancing therapeutic outcomes. With continued research and technological advancements, we anticipate that cellular therapy will improve both the quality of life and long-term health outcomes for patients.
